# HIV-1 Infection Causes a Down-Regulation of Genes Involved in Ribosome Biogenesis

**DOI:** 10.1371/journal.pone.0113908

**Published:** 2014-12-02

**Authors:** Claudia L. Kleinman, Margherita Doria, Elisa Orecchini, Erica Giuliani, Silvia Galardi, Nicolas De Jay, Alessandro Michienzi

**Affiliations:** 1 Lady Davis Institute for Medical Research, Sir Mortimer B. Davis-Jewish General Hospital and Department of Human Genetics, McGill University, 3755 Côte Ste-Catherine Road, Montréal, Quebec, H3T 1E2, Canada; 2 Laboratory of Immunoinfectivology, Bambino Gesù Children's Hospital, IRCCS, Rome, Italy; 3 Department of Biomedicine and Prevention, University of Rome “Tor Vergata”, Rome, Italy; University of British Columbia, Canada

## Abstract

HIV-1 preferentially infects CD4^+^ T cells, causing fundamental changes that eventually lead to the release of new viral particles and cell death. To investigate in detail alterations in the transcriptome of the CD4^+^ T cells upon viral infection, we sequenced polyadenylated RNA isolated from Jurkat cells infected or not with HIV-1. We found a marked global alteration of gene expression following infection, with an overall trend toward induction of genes, indicating widespread modification of the host biology. Annotation and pathway analysis of the most deregulated genes showed that viral infection produces a down-regulation of genes associated with the nucleolus, in particular those implicated in regulating the different steps of ribosome biogenesis, such as ribosomal RNA (rRNA) transcription, pre-rRNA processing, and ribosome maturation. The impact of HIV-1 infection on genes involved in ribosome biogenesis was further validated in primary CD4^+^ T cells. Moreover, we provided evidence by Northern Blot experiments, that host pre-rRNA processing in Jurkat cells might be perturbed during HIV-1 infection, thus strengthening the hypothesis of a crosstalk between nucleolar functions and viral pathogenesis.

## Introduction

CD4^+^ T cells, the primary cellular target of HIV-1, are progressively depleted during the course of infection, in a process by which the virus takes over the host cell machinery to successfully replicate. Therefore, it is of great interest to understand the complex relationship between HIV-1 and its host CD4^+^ T cell. Recent advances in high-throughput sequencing technologies, allowing for a detailed quantification of different aspects of gene expression at the genome-wide scale [1], [2], [3], provide an unprecedented opportunity to further understand this complex relationship. We present here a comprehensive transcriptomic analysis of polyadenlylated RNAs isolated from infected and mock Jurkat cells, followed by pathway analysis of the deregulated genes. At the transcriptional level, and in addition to the known expression changes related to infection, we found a marked down-regulation of genes functionally associated with the nucleolus.

The nucleolus is a sub-nuclear compartment that was originally described as the “Ribosome Factory” [4]. It is in this cellular compartment that the tandemly repeated clusters of genes encoding for ribosomal RNAs (rRNA) are localized, and this is the place where ribosomal RNA synthesis, pre-rRNA processing and ribosome subunits assembly occur [4]. In addition, it has been recently shown that hundreds of proteins involved in other cellular processes including cell cycle control, stress response, DNA damage response and repair, senescence and telomerase function and aging also accumulate in the nucleolus [4]. Moreover, the nucleolus is also the target of several viruses as a key site for their replicative process [4].

We found that the genes functionally associated with the nucleolus and down-regulated in HIV-1 infected cells encode for proteins mainly involved in regulation of the different steps of ribosome biogenesis, thus leading to the hypothesis that this process could be impaired during viral infection. These results were recapitulated in primary T CD4^+^. In addition, by Northern Blot analysis of the total RNA isolated from the mock- and HIV-1-infected Jurkat cells, we were able to show an alteration of the normal pre-rRNA processing pathway in the infected cell samples. In particular, we observed a dramatic decrease in the 30S transcript in the infected samples when compared to controls. Altogether, our study sheds new light into the intricate relationship between the host cell machinery and the infecting virus.

## Results

### HIV-1 infection globally alters gene expression profiles in CD4^+^ T cells

To characterize the effects of HIV-1 on host gene expression, Jurkat T cells (CD4^+^ T lymphoblastoma cell line, clone E6.1) were exposed or not to HIV-1 (NL4-3 strain) at a multiplicity of infection (m.o.i.) of 0.6, thus achieving after 48 hr maximal infection efficiency (∼70%), as determined by measuring the percentage of cells expressing the p24 Gag capsid antigen ([5] and data not shown), with a ∼85% cell viability as evaluated by trypan blue exclusion. In two independent infection experiments, both infected and mock-infected cells were collected at 48 hr post-infection and total RNA was isolated, subjected to poly(A) selection followed by reverse transcription, generation of cDNA libraries, and sequencing.

To gain a sequencing power allowing detection of HIV-1 associated changes, technical and biological replicates were sequenced at high coverage, resulting in an average of 150 millions paired-end reads per sample. We found that 3% of the total mapped reads corresponded to viral RNA.

In addition to the sequencing data generated for this study, and as an independent validation evidence for the results obtained, we analyzed the raw sequencing data from a previously published transcriptomic analysis [1], subjecting this dataset to the exact same pipelines applied to our samples. This dataset, obtained at 12 hr and 24 hr post-infection of the SupT1 T cell line with the LAI strain of HIV-1, will be referred to as Chang dataset (CHDT dataset) in what follows. In these samples, the percentage of total mapped reads corresponding to HIV sequences was higher, reaching 18% and 38% at 12 hr and 24 hr post-infection, respectively.

**Figure 1:**
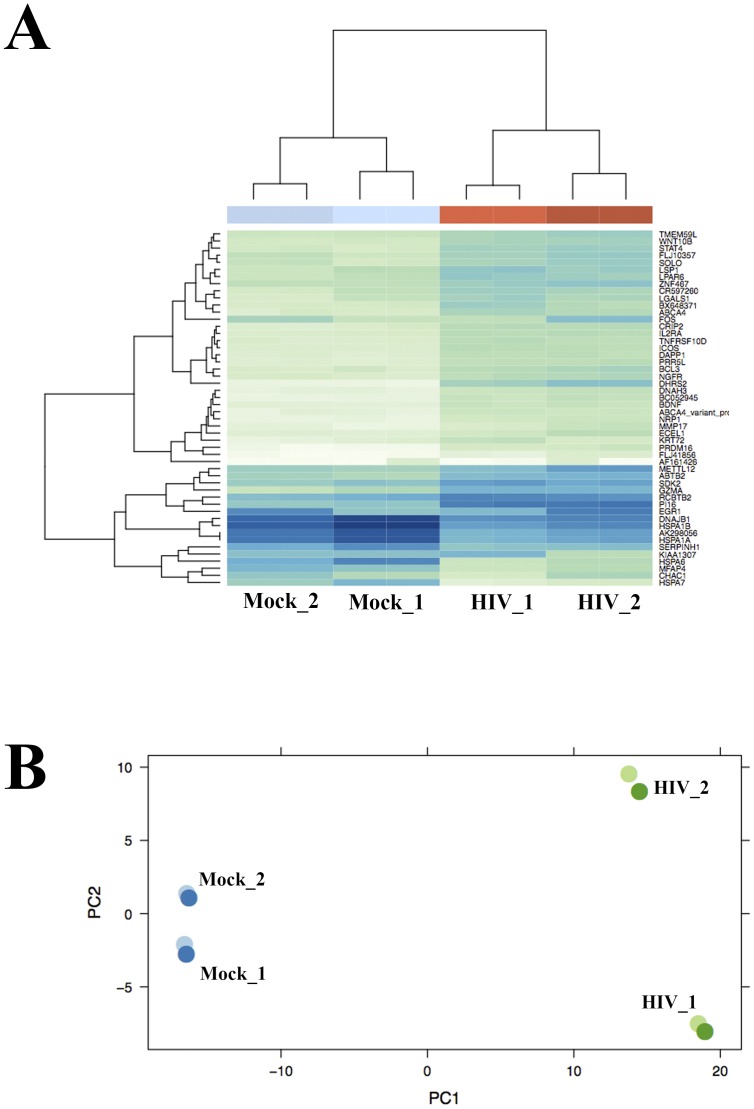
Viral infection globally alters gene expression profiles in CD4^+^ T cells. (A) Hierarchical clustering by gene expression using the 50 most variant genes. The agglomeration method used was complete linkage, with Euclidean distance. (B) PCA analysis shows a clear separation between mock and infected cells.

Globally, we found that gene expression profiles are largely impacted by viral infection: infected cells clearly separate from mock-infected cells in hierarchical clustering and principal component analysis (Figure 1). As expected, technical (sequencing) replicates show very similar expression profiles, while biological replicates display slightly larger differences. Variance-ranked clustering by expression is very robust, with infected cells separating from mock-infected cells regardless the number of genes considered, from 50 genes (Figure 1) to 1,000 genes (Figure S1).We detected significant change in expression levels (at FDR = 0.1, Table S5) for 10% of the 18,382 expressed genes in our samples.

When comparing the samples sequenced for this study to those belonging to the CHDT dataset, we found that the overall expression profiles differed (Figure S1). This is not unexpected, due to the difference in cell types, viral strains, infection strength, experimental design and sequencing technology used. In spite of these differences, however, the statistically significant changes between mock and infected cells observed in the two studies display a remarkable agreement, with almost half of the genes differentially expressed in our samples being also significantly deregulated in the CHDT dataset at 24 hr post-infection. This observation supports the biological relevance of the results obtained, for which changes in expression are related to viral infection and not affected by experimental variables.

Notably, we observed an overall trend towards induction of genes after infection. We found between 2 and 5 times more up-regulated genes than down-regulated ones, independently of the significance threshold selected (Table 1). The distribution of log ratios is skewed to the right (Figure S2, skewness = 1.1, D'Agostino test p-value<2.2e-16), with an excess of genes showing induction upon infection, indicating that the cells are, globally, more transcriptionally active after viral exposure.

**Table 1:** Summary of differentially expressed host genes in HIV-infected cells.

Criteria for selection	Downregulated genes	Upregulated genes	Ratio
FDR<0.01	169	485	1∶3
FDR<0.01 Fold Change>2	88	440	1∶5

In order to gain further insight on the specific categories of genes with affected expression, we next selected for analysis the expressed genes significantly and strongly deregulated (i.e. FDR<0.01 and fold change >2) and performed annotation and pathway analysis (The Database for Annotation, Visualization and Integrated Discovery or DAVID, [6], [7]). Among the 440 strongly up-regulated genes, 36 encode for proteins with well-characterized interactions with HIV-1 (Table S1). Among them we found *LGALS1* gene, encoding for a host soluble beta-galactoside-binding lectin that facilitates both virion binding and the infection of target cells [8], *IL-10* gene that encode for a immunoregulatory cytokine that facilitate viral persistence [9], and *CD69*, an early marker of CD4^+^ T cell activation [10]. Previously identified gene clusters related to transcription and transcription factor activity, apoptosis, cell motility and cell proliferation were also found, as expected, to be enriched in this set of up-regulated genes.

In contrast, only 88 cellular genes were found significantly down-regulated in infected samples. Functional annotation clustering of these genes by DAVID resulted in only few groups of genes with similar function (Table S2), with the most significant cluster corresponding to genes related to the nucleolus and organelle lumen, a category of genes that has so far gone unnoticed in previous studies on HIV-1.

The nucleolus is the ribosome factory of the cells, but it also plays a role in other cellular processes, including cell cycle control and cellular stress response [4]. The nine annotated genes we found associated to the nucleolus and deregulated (*DDX21*, *DHX33*, *HEATR1*, *RRS1*, *WDR43*, *GRWD1*, *NOM1*, *POLR1B* and *UTP20*) encode for proteins involved or supposed to be involved in the regulation of the ribosome biogenesis, from pre-rRNA transcription and processing to ribosomal RNP assembly and export. In particular, *POL1RB*, *HEATR1*, *WDR43*, *UTP20* and *DHX33* gene products play a role in rRNA transcription [11], [12], [13]. *POL1RB* gene product is one of the subunit of the RNA polymerase I, HEATR1 and WDR43 are t-UTP proteins (UTP10 and UTP5 respectively), with a proven involvement in both transcription and U3 snoRNA-dependent pre-rRNA processing [11]. *UTP20* encodes for a protein that may affect the acetylation of UBF thus activating RNA Pol I transcription [12], and DHX33 protein participates in rRNA transcription by increasing the association of Pol I with rDNA loci [13]. The *rss1* gene product in yeast is required for maturation of the 25S rRNA and has an essential function in the assembly of the 60S ribosomal subunit [14]. The human RRS1 protein was found in a proteome analysis of nucleolar extract suggesting that its role in ribosome biogenesis could be conserved [14], [15], [16]. Finally, *DDX21*, *NOM1* and *UTP20* gene products are well known to play a critical role in pre-rRNA processing [17], [18], [19].

**Figure 2:**
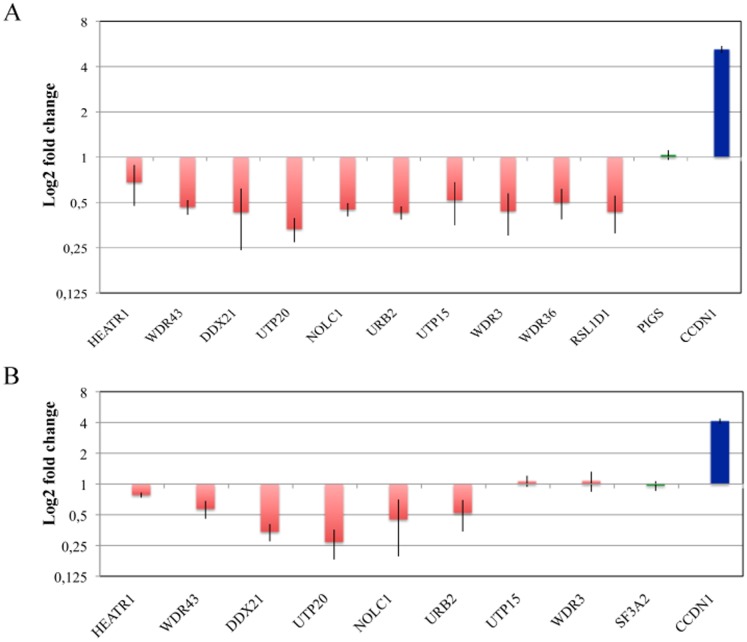
Validation of RNA-Seq experiments by RT-qPCR. RT-qPCR was used to validate the RNA sequencing data using total RNA isolated from mock-infected and HIV-1-infected Jurkat (A) and primary CD4^+^ T cells (B). Mean ± SD values obtained in three (A) or two (B) independent experiments are shown. Red, blue, and green bars depict genes that are down-regulated, up-regulated, or unaffected by HIV-1- infection, respectively, based on RNA-seq analysis.

We analyzed the expression of 10 genes representative of the ribosome biogenesis pathway (HEATR1, WDR43, DDX21 and UTP20 described above and other 6 genes found down-regulated in the RNA-seq analysis at lesser extent) by RT-qPCR using total RNA isolated from, mock-infected and infected samples (the same RNA employed in the RNA-seq and RNA isolated from other two independent infection experiments) and confirmed the decrease in the accumulation of the mRNA level of these genes associated with the nucleolus upon infection with HIV-1 (Figure 2a). Furthermore, we validated these results by applying RT-qPCR to the analysis of gene expression pattern of HIV-1 infected and mock-infected primary CD4^+^ T cells at day 3 post-infection (Fig.2b). Strikingly, in agreement with the results obtained in Jurkat cells, we found that 6 out of the 8 genes analyzed showed a decrease in their mRNA level in the infected primary CD4^+^ T cells at the time point analyzed. Two additional genes were tested, RLSD1 and WDR36, but these were expressed at very low level in primary CD4^+^ T cells (data not shown) and thus were excluded from this analysis.

In summary, despite the difference in the two experimental systems employed, Jurkat cells and primary CD4^+^ T cells, a similar pattern of expression for genes involved in the ribosome biogenesis can be observed. Altogether, these observations strongly support our hypothesis that, during HIV-1 infection, an impairment of the ribosome biogenesis might occur.

**Figure 3:**
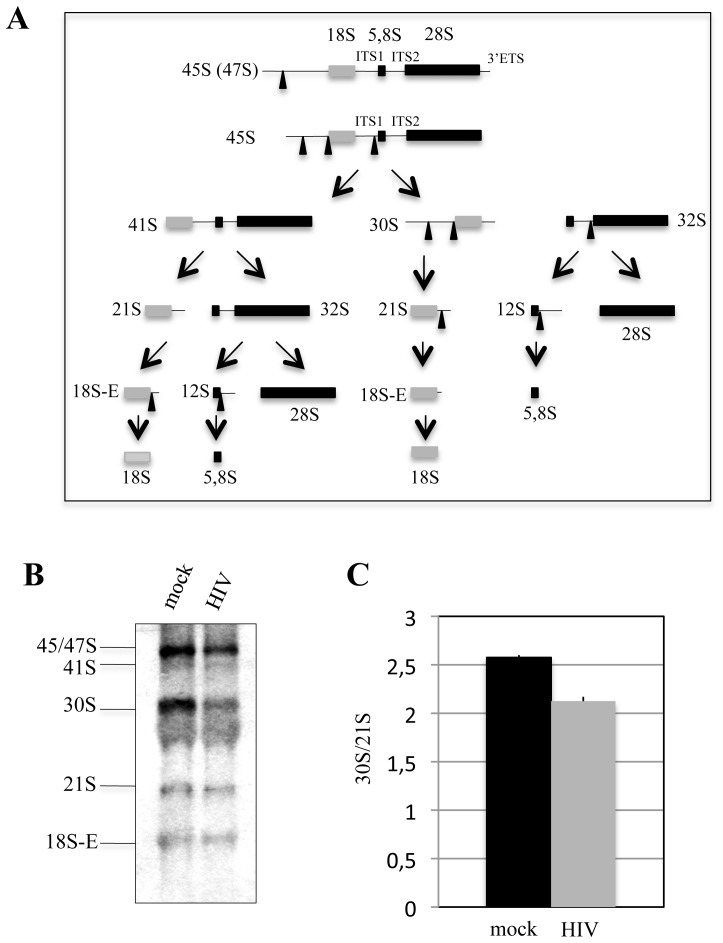
HIV-1 infection affects pre-rRNA processing. (A) Schematic representation of the pre-rRNA processing in mammals [50]. (B) Northern Blot analysis of total RNA isolated from infected Jurkat cells and mock cells using an ITS1 rRNA specific probe. (C) Quantitation of the signals of Northern experiments reported as ratio between 30S and 21S rRNA precursors in infected Jurkat cells as compared with mock cells. The amounts of the two species were calculated after Northern blotting by phosphoimaging. This analysis was performed on both the sets of RNA analyzed by RNA-seq and standard deviation in the ratio 30S/21S is indicated.

To confirm this hypothesis we assessed whether the pre-rRNA maturation process, one of the steps of the ribosome biogenesis, is affected by viral infection. To this aim, a Northern Blot analysis of total RNA isolated from the mock and infected Jurkat cells was performed (a fraction of the same total RNA used for the RNA-seq analysis) using specific probes for the Internal Transcribed Spacer 1 (ITS1) rRNA sequences (Figure 3a; Figure 3b) and for GAPDH mRNA as loading control (data not shown). We found a substantial reduction in the accumulation of the 30S pre-rRNA in infected samples compared to controls (Figure 3a; Figure 3b). The densitometric quantitation of the Northern Blot signals was reported as the ratio between 30S and 21S rRNA precursors, showed a reduction of this ratio of about 20% in the infected samples compared to mock-infected samples (Figure 3c), while no difference in loading of the RNA samples was observed by using a GAPDH specific probe (data not shown).

**Figure 4:**
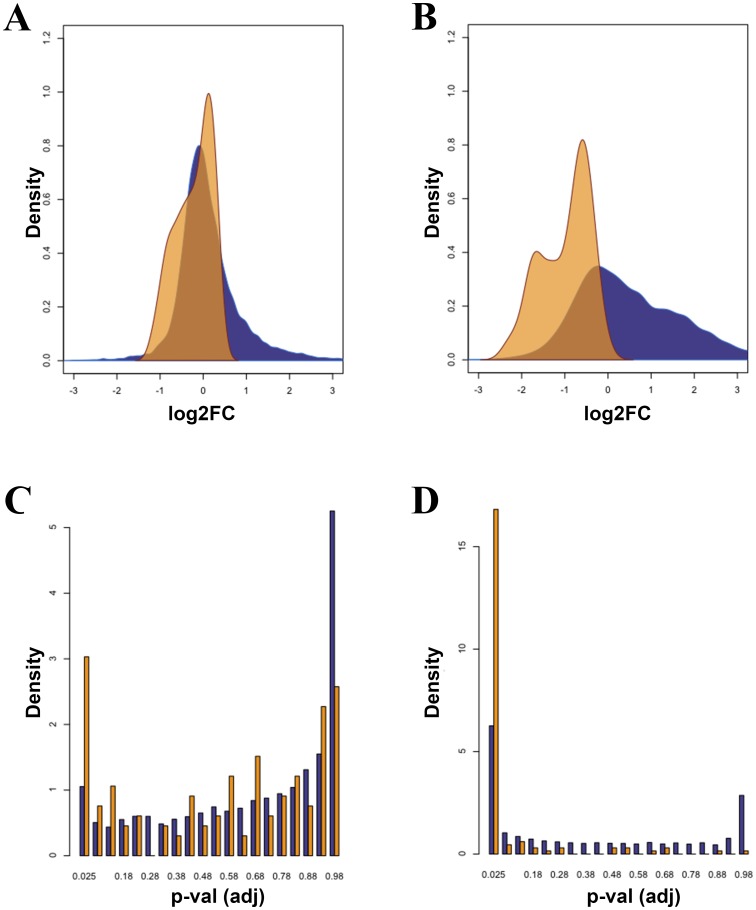
Genes encoding for proteins regulating Ribosome Biogenesis are negatively affected by viral infection. (A) and (B) Distribution of log ratios/fold-change (FC) in expression levels for all genes (blue) and genes encoding for nucleolar proteins involved in the ribosome biogenesis (orange) using Kernel density estimation; (A) samples sequenced in this study; (B) samples obtained from CHDT dataset, 24hs post infection. (C) and (D) Distribution of p-values, adjusted for multiple testing, for all genes (blue) or genes involved in the biogenesis of the nucleolus (orange); (C) this study, (D) from CHDT dataset.

To further analyze the effect of HIV-1 infection on ribosome biogenesis, we compiled a list of all genes encoding for proteins present in the nucleolar proteome of Jurkat cells [20], with either suggested or demonstrated role in the ribosome biogenesis (Table S3). In striking contrast to the overall trend towards induction observed for all genes upon infection (Figure S2; Table 1), genes involved in ribosome biogenesis were markedly down-regulated (Figure 4). Although, individually, not every gene reached statistical significance in the differential expression analysis, the overall distribution of genes related to ribosome biogenesis was very different from the general distribution of all other genes (Figure 4a, t-test P-value<2e-16), indicating a trend towards down-regulation. The same result, if not stronger, was obtained with the CHDT dataset (Figure 4b): only one out of 132 genes has very slightly increased expression after infection (*KIAA1398*), while all other genes were down-regulated. In contrast, no significant effect was observed at 12hs post-infection (Figure S3). Similarly, the less pronounced effect observed in our samples is probably due to the lower efficiency of HIV-1 replication, but remains significant: none of the 56 genes with increased expression (log2FC>0) reached statistical significance (FDR<0.05). Analogously, none of the ribosomal proteins showed statistically significant up-regulation at the mRNA levels. On the contrary, 20 genes involved in ribosome biogenesis were significantly down-regulated (FDR<0.05, log2FC<0), with five of them showing less than half the expression level after infection: *DDX21*, *HEATR21*, *RRS1*, *UTP20*, *WDR43*. The proportion of significantly deregulated genes among the nucleolar biogenesis cluster was much higher than for all genes (Figure 4c; Figure 4d). Overall, these results bring to light a novel mechanism of host gene expression regulation mediated by HIV-1 infection, involving the impaired synthesis of functional ribosomes.

## Discussion

Transcriptome sequencing by RNA-Seq produces a highly multidimensional type of data, allowing studies on simultaneously different aspects of gene expression, transcription and mRNA processing at a genome-wide scale. In this study, we focused on ribosome biogenesis, a process that we showed may be largely altered during viral infection. Ribosome biogenesis is a highly regulated multistep process that starts with pre-rRNA transcription within the nucleolus and ends with the formation of functional ribosomes in the cytoplasm [4]. Viral interactions with the nucleolus, sometimes disrupting nucleolar function, have been documented before for several viruses [21]. Upon infection, many viral and/or cellular proteins transit through the nucleoli of the cells, and numerous host nucleolar proteins are redistributed to other cellular compartments [21]. In this regard, in the early stages of infection, Newcastle disease virus (NDV) matrix (M) protein accumulates in the nucleolus of the host cells by binding the B23 nucleolar phosphoprotein and this interaction facilitate NDV replication [22]. Upon infection with herpes simplex type 1 (HSV-1), profound alterations of nucleolar morphology of the host cell occur. Nucleolin, B23 and UBF proteins leave the nucleolus to accumulate into the viral DNA replication centres (VRCs) [23], [24], [25], [26]. In addition, ribosomal protein L9 interacts with the mouse mammary tumor virus (MMTV) Gag protein in the nucleolus of the cells and knockdown of the endogenous L9 cause an impairment of virus production [27]. These results lead to the hypothesis that efficient MMTV particle assembly is dependent upon the interaction of Gag and L9 in the nucleoli of infected cells [27]. Recently, the use of proteomic analysis of cells either infected with different viruses or stably expressing specific viral proteins allowed to identify changes in nucleolar composition that are of functional relevance to the infection [28], [29], [30], [31]. In general, the role of viral perturbation of protein localization is not completely elucidated, but it has been shown to affect different steps of viral replication and various cellular processes, such as transcription, post-transcriptional processing and cell cycle control [21]. Concerning HIV-1, it was previously reported that the viral regulatory proteins Tat and Rev are both mainly localized in the nucleolus [21]. We have demonstrated that nucleolar localization of Tat and Rev, as well as the trafficking of some viral transcripts through this sub-cellular compartment, is critical for viral replication [32], [33], [34]. Furthermore, it has been recently demonstrated that a subpopulation of Gag polyprotein of HIV-1 traffic trough the nucleolus during viral replication suggesting that in this nuclear compartment could contribute to HIV-1 RNA assembly and packaging [35]. In addition, a quantitative proteomic analysis of the nucleolar composition of Jurkat cells stably expressing the HIV-1 Tat protein has shown that the expression of this viral protein causes changes in abundance of specific host nucleolar proteins which may reflect a viral strategy to facilitate viral production [31].

Overall, these observations provide a link between HIV-1 replication and nucleolus.

In the work presented here, by analyzing expression levels of all host genes in HIV-1-infected cells by RNA-seq, we identified nine genes functionally associated with the nucleolus and whose expression was down-regulated in infected samples. A closer look at these genes showed that they all encode for proteins that play critical roles in ribosome biogenesis. Down-regulation of genes involved in ribosome biogenesis was validated by RT-qPCR analysis using total RNA of Jurkat cells. Moreover, these results were further confirmed in infected primary CD4^+^ T lymphocytes. Importantly, the same effect was observed in independent RNA-Seq datasets (CHDT dataset; [3]), where we found an even larger number of down-regulated genes with the same functional role and cellular localization. By performing a Northern Blot analysis using the same total RNA subjected to the RNA-seq experiments, we were able to show an impairment of pre-rRNA processing in HIV-1-infected samples compared to mock-infected samples, resulting in a marked decrease in the accumulation of the 30S rRNA precursor. These results demonstrated that, indeed, HIV-1 infection affects at least one of the steps leading to ribosome biogenesis, uncovering a novel mechanism of regulation of host gene expression mediated by the virus.

In support of this hypothesis, it has been previously reported that other viruses can either promote, as hepatitis C virus (HCV) [36], or inhibit, as poliovirus [37], the host pre-rRNA synthesis and, notably, herpes simplex virus type 1 (HSV-1) can affect pre-rRNA processing without affecting pre-rRNA transcription [38]. Concerning HIV-1, it has recently been reported that expression of the viral Tat protein in Drosophila oocytes leads to a dramatic reduction of cytoplasmic ribosomes, probably caused by an impairment of pre-rRNA maturation [39]. The Nef protein, in turn, has been found associated to components of the 40S ribosomal subunit, in particular the 18S rRNA and the RPS10 protein [40]. In addition, it was previously published that HIV-1 infected CEMx174 T cells displayed a subtle decrease in soluble and membrane-associated polyribosomes compared to mock-infected control [41]. Moreover, infected cells showed suppression of host mRNAs translation mediated by the action of the viral protein Vpr while the translation of viral structural protein is sustained [41].

In light of our results, it can be hypothesized that alteration of ribosome biogenesis mediated by HIV-1 may allow the virus to alter the protein translation machinery or to induce stress signals, thus inhibiting host protein synthesis and, ultimately, inducing apoptotic pathways. This HIV-1 strategy will most likely come into action in the late steps of viral replication, once progeny virions have been massively produced and cell survival has become dispensable, if not deleterious, for the virus. Further studies will be necessary to dissect the link between ribosome biogenesis alteration and viral infection, possibly unraveling a novel intricate network of interactions between HIV-1 and host cell.

## Materials and Methods

### Cells

Jurkat cells (clone E6.1; ATCC) and primary CD4^+^ T lymphocytes were maintained in RPMI 1640 medium, supplemented with 10% heat-inactivated fetal bovine serum, 2 mM L-glutamine, 100 U/ml penicillin-streptomycin (Euroclone and Gibco-BRL). 293T cells were maintained in Dulbecco's modified Eagle's medium supplemented as above. All reagents were from Euroclone. Peripheral blood mononuclear cells (PBMCs) were obtained by Ficoll separation of buffy coats from healthy donors. Primary CD4^+^ T cells were isolated from PBMCs by negative selection with EasySep Human CD4^+^ T Cell Enrichment Kit (STEMCELL). Prior RNA extraction, HIV-infected T lymphocytes cultures were depleted of non-infected CD4^+^ cells using immunomagnetic beads (Dynabeads CD4, Dynal, Invitrogen) according to manufacturer's instructions.

### HIV-1 infection

Stocks of VSV-G-pseudotyped HIV-1 were prepared by transfecting 20 µg of proviral plasmid (pNL4-3; Cat. N. 114, NIH AIDS Research and Reference Reagent Program) and 3.5 µg of pCMV-VSV-G (kind gift of Dr. M. Federico) into 293T cells using the standard calcium phosphate method. At 48 hr post-transfection, cell culture supernatants were collected, clarified and stored at −80°C. Viral stocks were titrated by anti-p24 enzyme-linked immunosorbent assay (ELISA) according to manufacturer's instructions (Innogenetics) and viral infectivity was evaluated with CEM-GFP indicator cells as described elsewhere [49].

For HIV-1 infection of Jurkat cells, a previously described procedure was used [42]. Specifically, Jurkat cells were resuspended at 2 5 10^6^ cells/ml in medium with 8 µg polybrene/ml, either alone or together with 100 ng of p24/10^6^ cells (0.6 m.o.i.), and centrifuged at 2500 rpm for 90 min at 30°C. Subsequently, cells were washed and resuspended in medium at 7 5 10^5^/ml.

To infect primary CD4^+^ T lymphocytes, cells were resuspended at 10×10^7^ cells/ml in medium with 8 µg Polybrene/ml either alone or together with 50 ng of p24/10^6^, centrifuged at 2,800 rpm for 30 min at 30°C, then incubated for 2 h at 37°C with gentle mixing every 30 min. Then, cells were washed and cultivated at 5×10^6^/ml in medium supplemented with 100 ng/ml of *Staphylococcus aureus* enterotoxin B and 100 IU/ml of human recombinant interleukin-2 (IL-2; both from Sigma-Aldrich) for 3 days before analysis. Mock-infected cells were treated like infected cells but in the absence of virus.

### RNA sequencing

At 48 hr post-infection, HIV-1-infected and mock-infected Jurkat cells were collected by centrifugation and total RNA isolated using TRizol (250 µl/10^6^ cells; Invitrogen). Total RNA was cleaned-up using column of the miRNeasy kit (including a DNaseI treatment step) and quality assessed by Agilent 2100 BioAnalyzer (Agilent Technologies). Libraries for RNA-sequencing were prepared from 2ug RNA per sample, using the Illumina TruSeq RNA Sample Preparation Kit v2, following manufacturer's instructions. Samples were indexed 2 per lane and sequenced with 100bp paired end reads in an Illumina HiSeq 2000 sequencer. Sequencing runs were processed by Illumina's CASAVA 1.8 software.

Reads were trimmed with custom scripts, removing low quality bases at end of reads (phred33<30) and clipping Illumina adapter sequences and three bases at the 5′ end of each read. Reads shorter than 30 bp after trimming were discarded. The resulting high quality RNA-seq reads were aligned to the human reference genome build hg19 using Tophat v1.4.1 [43] coupled with Bowtie v0.12.8 [44]. Reads mapping to multiple (more than ten) locations were discarded for downstream analysis. We then mapped the remaining unmapped reads to the HIV genome (GenBank accession number AF324493.2) to compute the percentage of viral reads present in the samples.

Integrative genomics viewer (http://broadinstitute.org/igv) was used for visualization. Multiple quality control metrics were obtained using FastQC (http://www.bioinformatics.bbsrc.ac.uk/projects/fastqc), SAMtools [45], BEDtools [46] and custom scripts (Table S4). Bigwig tracks for visualization were generated with custom scripts, using BEDtools and UCSC tools.

Raw sequencing data from [1] was downloaded from the NIH Gene Expression Omnibus database (accession code GSE38006) and processed with the pipeline described above to allow for comparisons with the samples sequenced in this study, using the corresponding HIV genome as reference (GenBank accession no. K02013).

### Analysis of gene expression

To estimate gene expression levels, we used all exonic reads mapping within the maximal genomic locus containing each gene and its known isoforms, normalizing by library size using DESeq version 1.16.0, Bioconductor release 2.14 [47]. For clustering, correlation and principal component analysis, variance-stabilized expression values were derived using DESeq. Hierarchical clustering was performed using euclidean distance and complete linkage as the agglomeration method. Differential expression analysis was performed using DESeq, where statistical significance is calculated using the negative binomial distribution as a null distribution for gene expression values, with variance and mean estimated from the data and linked by local regression. Normalized expression levels obtained for all coding genes can be found in Table S5.

### Northen Blot Analysis

Total RNA was electrophoresed in a 1.2% agarose-6.8% formaldehyde gel and blotted onto a nylon membrane. Hybridization was performed with a radiolabeled probe complementary to ITS1 rRNA sequence (5′- GCTCCTCCACAGTCTCCCGTTAATGATC-3′). A probe specific for GAPDH mRNA was used as loading control.

### Quantitative reverse transcription (qPCR)

Total RNA isolated using TRIzol reagent (Invitrogen) was reverse transcribed using M-MLV RT (Invitrogen) following the manufacture instructions and the resulting cDNA (25ng) used for qPCR analysis.

The qPCR analysis was performed using the SYBR Green PCR master mix (Life Technologies) and the following primers:

HEATR1 for: 5′- ACTTGTCGCCTTACTTCCTG -3′

HEATR1 rev: 5′- ATTCTTGTCTCGTGGTATGGC -3′

WDR43 for: 5′- GGAACGTACAGACATGCAAAG -3′

WDR43 rev: 5′- GGCTGATACATAGGGAACTGAC -3′

UTP20 for: 5′- GAAGCATTCCACTTTGACCAC -3′

UTP20 rev: 5′- CTTCTCTTCCTCATCCACACG -3′

DDX21 for: 5′- CTGGGTGTTTGCTTTGATGTAC-3′

DDX21 rev: 5′- AGTTCTGGTTGCTCTGTGG -3′

SF3A2 for: 5′- AACTCTGCCTGACACTTCAC -3′

SF3A2 rev: 5′- CACCTCCACCTTGACCTTC -3′

NOLC1 for: 5′- TTCAGACCCCTAACACATTTCC -3′

NOLC1 rev: 5′- GCTTGGCATCAAAGGAGTTG -3′

URB2 for: 5′-GAGTTTGCTGTGTTTTCCCC -3′

URB2 rev: 5′- GAGGTCCAGGATGAGGTAAATG -3′

UTP15 for: 5′- GGTCGGGATGAGAAGGAAATC -3′

UTP15 rev: 5′- GGACTGACCAATTACAGGCAG -3′

WDR3 F for: 5′- GGGAGATGGAAAGAGAAGCAG -3′

WDR3 rev: 5′- CAATAGCCTCCATAATCCTCTCAG -3′

WDR36 F for: 5′- AGGATGGAAAGTTGGAGTGAC -3′

WDR36 rev: 5′- CCCCAGTCTTGACGAAACTTC -3′

RSL1D1 F for: 5′- ACGTTGGAATGCAAATTGAGC -3′

RSL1D1 rev: 5′- ACGAGGAAAAGATGGGAAGTG -3′

PIGS for: 5′- TCTTTGACTGAGGATGTGCTTG -3′

PIGS rev: 5′- GGGTCTGGGTTGAGTAAACTG -3′

Relative expression was calculated as log2 fold change of RT-qPCR data of HIV-1 over mock samples corresponding to ΔΔC_T_. ΔΔC_T_ was calculated using SF3A2 gene as a calibrator in Jurkat samples, as the expression of the SF3A2 did not significantly change between the mock and the infected samples in our RNA-seq data. *PIGS* gene was used as calibrator in primary CD4^+^ T cells samples as previously reported [3].

## Supporting Information

Figure S1 **Viral infection globally alters gene expression profiles in CD4^+^ T cells.** (A) Hierarchical clustering by gene expression using the 1,000 most variant genes. Euclidean distance was used as distance measure, complete linkage as the agglomeration method. LCL: lymphoblastoid cell lines [48] (B) PCA analysis of the CHDT dataset; only after 24hs infection the expression profiles are clearly distinct, while at 12hs the infected samples are closer to the Mock samples.
(TIFF)

Figure S2 **HIV infected cells are found in a transcriptionally active state.** Distribution of log ratios/fold change (FC) in expression levels genome-wide. The observed distribution is skewed to the right (skewness = 1.1), with an excess of genes showing induction upon infection (kurtosis = 8.1).
(TIFF)

Figure S3 **Genes related to the “nucleolus” cluster and involved in ribosome biogenesis are not significantly affected at 12hs post infection in the samples obtained from CHDT dataset.** (A) Distribution of log ratios/fold-change (FC) in expression levels for all genes (blue) and genes involved in the biogenesis of the ribosome (orange) using Kernel density estimation; (B) Distribution of p-values, adjusted for multiple testing, for all genes (blue) or genes involved in the biogenesis of the ribosome (orange).
(TIFF)

Table S1 **Up-regulated genes coding for proteins with well-characterized interactions with HIV-1.**
(XLSX)

Table S2 **Functional annotation clustering by DAVID of genes with significant (FDR<0.01) and strong (log2FC<-1) down-regulation with expression above a minimum level (normalized expression>100) in at least one of the samples.**
(XLSX)

Table S3 **Genes present in the nucleolar proteome of Jurkat cells** [20] **with a demonstrated or proposed role in ribosome biogenesis.**
(XLSX)

Table S4 **Sequencing statistics.**
(XLSX)

Table S5 **Normalized expression levels for all genes analyzed.**
(XLSX)
