# Supplementary figures and images for: HIV-1 Infection Causes a Down-Regulation of Genes Involved in Ribosome Biogenesis
Source: PLoS One. 2014 Dec 2;9(12):e113908. doi: 10.1371/journal.pone.0113908 (PMC4252078; doi:10.1371/journal.pone.0113908)

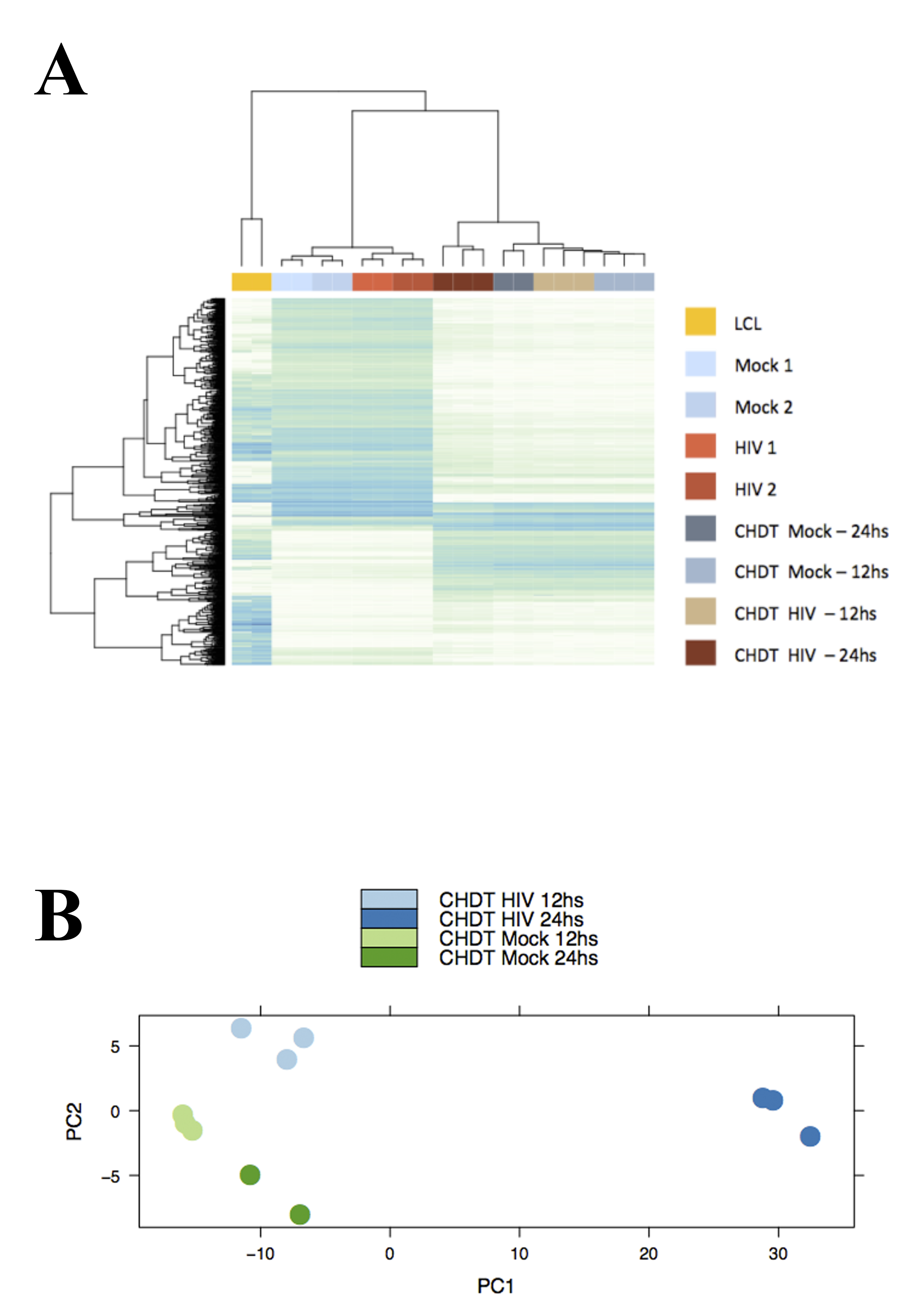

Supplement: Figure S1 — Viral infection globally alters gene expression profiles in CD4+ T cells. (A) Hierarchical clustering by gene expression using the 1,000 most variant genes. Euclidean distance was used as distance measure, complete linkage as the agglomeration method. LCL: lymphoblastoid cell lines [48] (B) PCA analysis of the CHDT dataset; only after 24hs infection the expression profiles are clearly distinct, while at 12hs the infected samples are closer to the Mock samples. (TIFF) [file pone.0113908.s001.tiff]

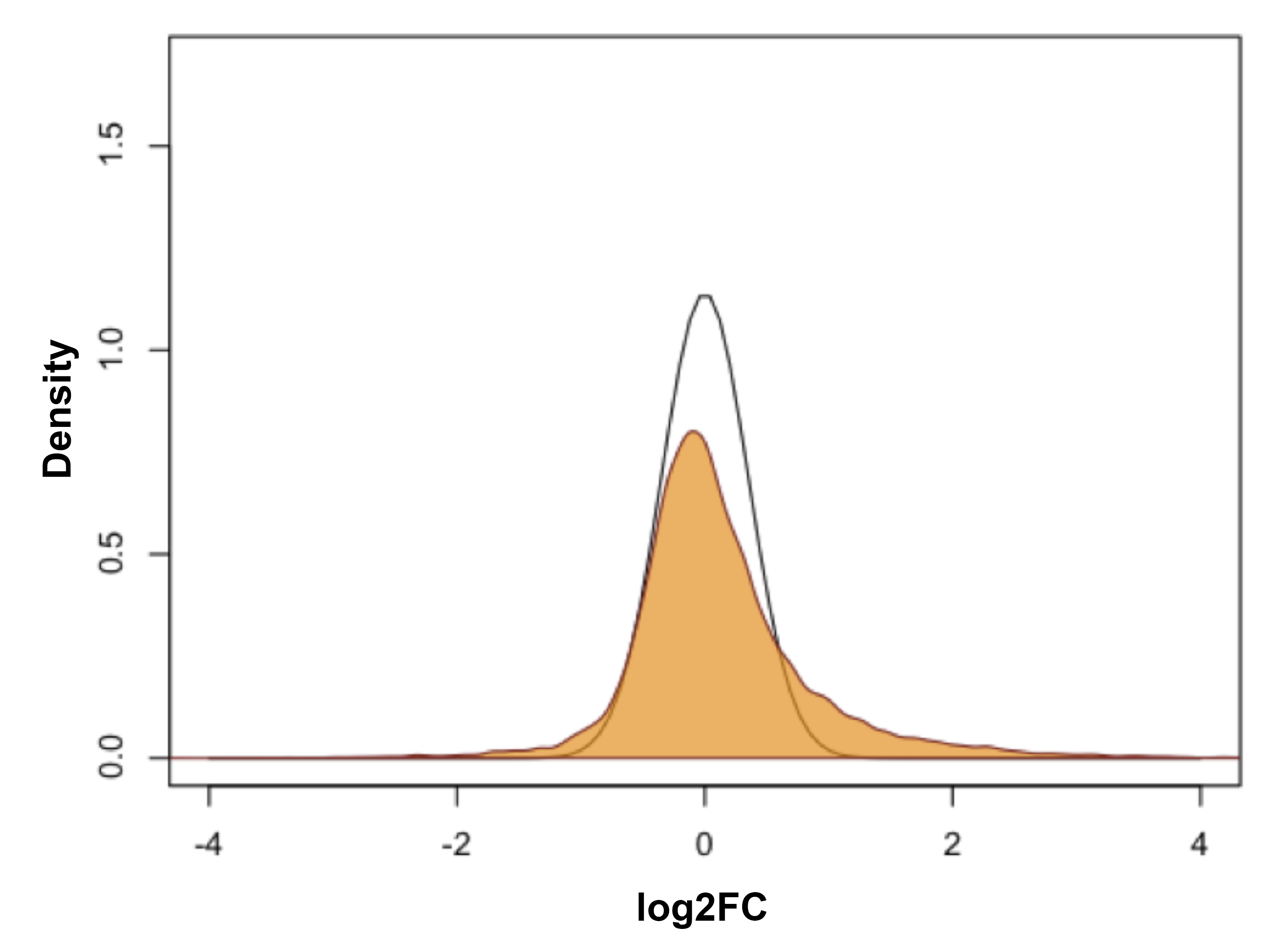

Supplement: Figure S2 — HIV infected cells are found in a transcriptionally active state. Distribution of log ratios/fold change (FC) in expression levels genome-wide. The observed distribution is skewed to the right (skewness = 1.1), with an excess of genes showing induction upon infection (kurtosis = 8.1). (TIFF) [file pone.0113908.s002.tiff]

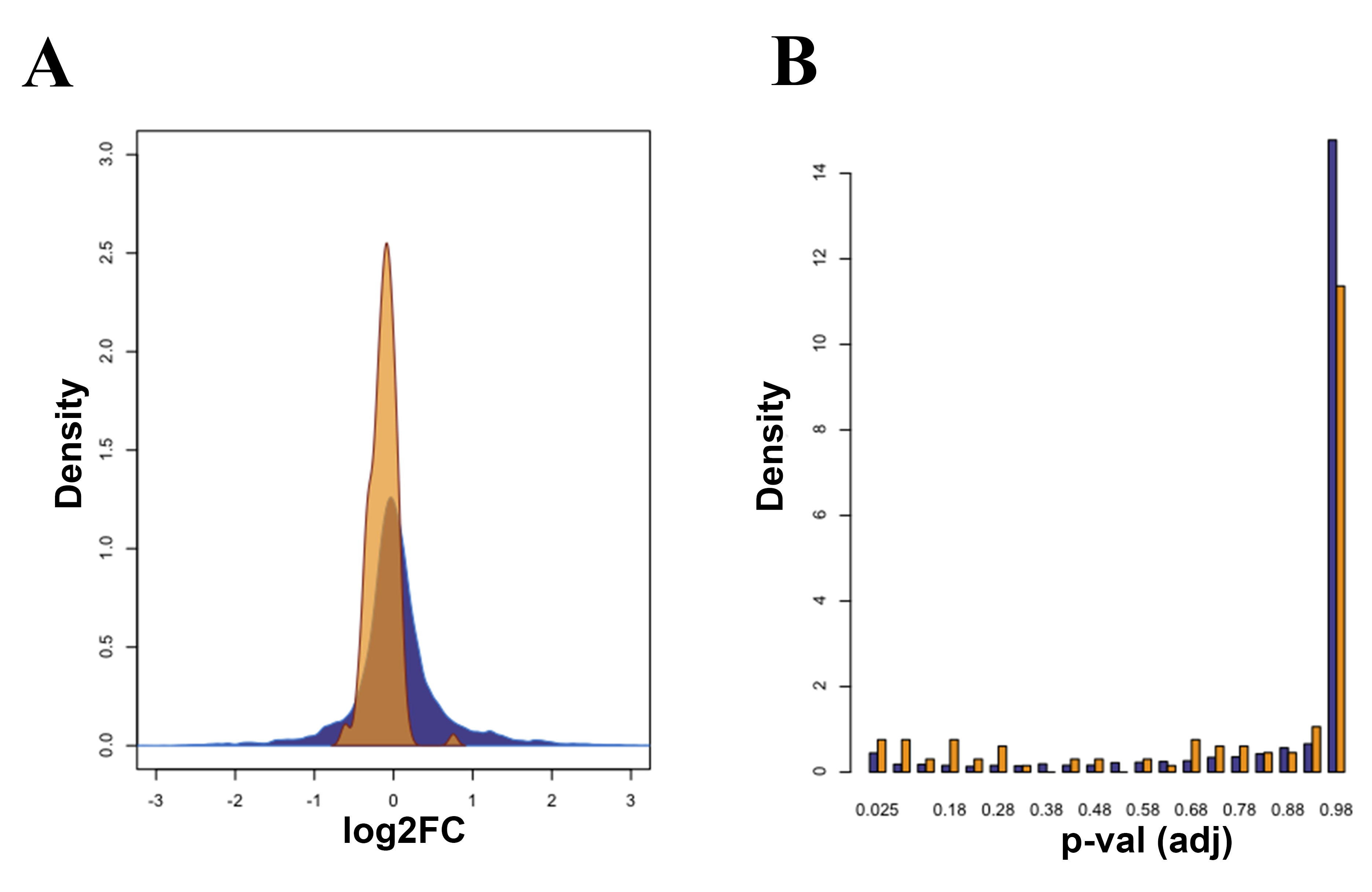

Supplement: Figure S3 — Genes related to the “nucleolus” cluster and involved in ribosome biogenesis are not significantly affected at 12hs post infection in the samples obtained from CHDT dataset. (A) Distribution of log ratios/fold-change (FC) in expression levels for all genes (blue) and genes involved in the biogenesis of the ribosome (orange) using Kernel density estimation; (B) Distribution of p-values, adjusted for multiple testing, for all genes (blue) or genes involved in the biogenesis of the ribosome (orange). (TIFF) [file pone.0113908.s003.tiff]
